# Nutmeg Essential Oil, Red Clover, and Liquorice Extracts Microencapsulation Method Selection for the Release of Active Compounds from Gel Tablets of Different Bases

**DOI:** 10.3390/pharmaceutics15030949

**Published:** 2023-03-15

**Authors:** Jurga Andreja Kazlauskaite, Inga Matulyte, Mindaugas Marksa, Jurga Bernatoniene

**Affiliations:** 1 Department of Drug Technology and Social Pharmacy, Medical Academy, Lithuanian University of Health Sciences, LT-50161 Kaunas, Lithuania; 2 Institute of Pharmaceutical Technologies, Medical Academy, Lithuanian University of Health Sciences, LT-50161 Kaunas, Lithuania; 3 Department of Analytical and Toxicological Chemistry, Medical Academy, Lithuanian University of Health Sciences, LT-50161 Kaunas, Lithuania

**Keywords:** essential oil, nutmeg, red clover, polyphenols, liquorice, spray-drying, freeze-drying, gel tablets, gelatin, pectin

## Abstract

The current study presents the most suitable method for encapsulating nutmeg essential oil with liquorice and red clover. Two widely used methods, spray-drying and freeze-drying, were employed to find the most suitable for essential oil volatile compounds’ protection. Results showed that freeze-dried capsules (LM) had a higher yield (85.34%) compared to the exact formulation of spray-dried microcapsules (SDM)—45.12%. All the antioxidant and total phenolic compounds’ results obtained with the LM sample were significantly higher compared with SDM. LM microcapsules were incorporated in two different bases with no additional sugar (gelatin and pectin) for targeted release. Pectin tablets had firmer and harder texture properties, while gelatin tablets had a more elastic texture. There was a significant impact on texture changes caused by microcapsules. Microencapsulated essential oil with extracts can be used alone or in a gel base (pectin or gelatin, depending on user preferences). It could be an effective product to protect the active volatile compounds and regulate the release of active compounds and give a pleasant taste.

## 1. Introduction

Microencapsulation has many applications in the food, cosmetic and pharmaceutical industries, such as protecting, isolating, or controlling a given substance’s release. Microcapsules as delivery systems offer numerous advantages over conventional dosage forms, including improved efficacy, patient compliance, and convenience. Changing a liquid into a powder enables a variety of different applications for substances [1,2]. Microcapsules are constructed from an encasing substance that surrounds a core containing the active ingredient. The final particle size can range from 0.2 to 5000 m in diameter depending on a number of variables, including processing and material selection [3]. For microcapsules’ preparation, various techniques can be employed, including spray drying, spray chilling or spray cooling, extrusion coating, fluidized-bed coating, liposomal entrapment, lyophilization, coacervation, centrifugal suspension separation, co-crystallization, and inclusion complexation [4].

Essential oil microencapsulation is needed to protect active compounds’ evaporation and oxidative decomposition, to mask strong odors, and in pharmaceuticals, to control release [2]. Additionally, plant extracts that contain active polyphenols can be encapsulated. These compounds sometimes needs to be microencapsulated because they have an unpleasant bitter taste or limited concentration that remains available following oral administration due to an insufficient gastric residence time, low permeability and/or solubility within the gut, and instability [4,5]. 

Emulsification, which affects encapsulation efficiency, powder characteristics, and storage stability, is a crucial step that should be performed before drying, particularly in the microencapsulation of essential oils, which requires the use of various wall materials. Different wall materials, including some carbohydrates, protect active substances against adverse temperatures, pH, humidity, oxygen, and other components that can react with the protected material [6]. 

One of the most used microencapsulation methods is spray-drying. This process is affordable, simple to alter and use, and results in a high-quality product [7]. The feed emulsion’s composition and drying parameters have an impact on the quality of spray-dried microcapsules. The coating substance can be altered and customized to deliver ideal features. Coverage of the active substance gives oxidation protection and wall materials with high solubility, good emulsification, film-forming, and drying capabilities in the finished microcapsules [8]. The main concern when spraying emulsions containing essential oils is the evaporation of volatile compounds due to the high spraying temperature. The researchers proved that it is possible to produce powders of heat-labile compounds even using 200 °C temperature; therefore, it should be safe to use a spray-drying process in preparation for essential oil microcapsules [9]. 

Freeze-drying (lyophilization) is a process that can protect product quality by limiting chemical reactions, and biological and microbiological deterioration [10]. It is particularly appealing for drying heat-sensitive and biologically active components because drying is carried out in a vacuum and at temperatures lower than the materials’ ambient temperature. Nevertheless, it has downsides like high energy consumption and long processing times [11,12,13]. 

Choosing the right inclusion material is a crucial step since encapsulating shields active volatile molecules from the environment, as was previously discussed. The increase in the stability of essential oil volatile compounds and the controlled delivery of encapsulated substances depends on the wall material. However, microcapsules after production often lack the desired effect, therefore incorporating microcapsules into pharmaceutical form can solve several problems.

Oral administration forms come in a variety of formats, such as tablets, soft and hard gel capsules, elixirs, suspensions, chewable tablets, etc. [14]. The latter form is becoming increasingly popular due to its convenient consumption in people with dysphagia, colorful appearance, smell, and because of a high sugar content pleasant taste. Most often, this form is found in food supplements [15]. Various polymeric materials are used for the formation of gels, such as gelatin, pectin, agar, and sugar is needed for the stabilization of their structure [16]. Nevertheless, natural substitutes can be used to form these gels, thus avoiding added sugar. 

This study aims to compare freeze drying with spray drying technology to preserve extracts and nutmeg essential oil and to choose the best microcapsules to incorporate in different base, sugar-free chewable tablets on a laboratory scale.

## 2. Materials and Methods

### 2.1. Plant Material and Reagents

*Myristica fragrans* seeds’ country of origin was Grenada (supplier Spaisvilė, Pašaltuonys, Lithuania). 

*Trifolium pratense* L. samples were collected in *Trifolium pratense* L. fields in Laičiai, Kupiškis district, Lithuania (latitude 55°53,024.2″ N; longitude 25°19,036.0″ E). *Glycyrrhiza glabra* L. roots (the country of origin is China) were bought from LSMU pharmacy (Kaunas, Lithuania). Dried herbs and seeds (blower buds and roots) were ground separately using an Ultra Centrifugal Mill ZM 200 (Retsch, Haan, Germany). 

Gum Arabic and maltodextrin were purchased from Sigma-Aldrich, (Steinheim, Germany). Ethanol (96%) used for extraction was purchased from Vilniaus degtinė (Vilnius, Lithuania). In this experiment, purified water was prepared with GFL2004 (GFL, Burgwedelis, Germany). Deionized water was prepared with Millipore, SimPak 1 (Merck, Darmstadt, Germany). The following reagents were used as standards, genistein, daidzein, and glycyrrhizin acid (Sigma-Aldrich, Steinheim, Germany). The 2,2′-azino-bis-(3-ethylbenzothiazoline-6-sulfonic acid) (ABTS), 2,2-diphenyl-1-picrylhydrazyl radical (DPPH), and β-CDs were purchased from Sigma-Aldrich (Hamburg, Germany); aluminum chloride, hexaethylenetetraamine, dimethyl sulfoxide (DMSO), acetic acid obtained from Sigma-Aldrich (Buchs, Switzerland); potassium persulfate obtained from Alfa Aesar (Karlsruhe, Germany); Folin–Ciocalteu’s phenol reagent (Merck, Darmstadt, Germany); monosodium phosphate, ferrous sulfate heptahydrate, saline phosphate buffer, and hydrogen peroxide obtained from Sigma-Aldrich (Schnelldorf, Germany); disodium hydrogen phosphate obtained from Merck (Darmstadt, Germany); alginic acid sodium salt from brown algae obtained from Sigma-Aldrich (Shanghai, China). Calcium chloride (Farmalabor, Pozzillo, Italy).

### 2.2. Microcapsules’ Formulation and Preparation

Ethanolic red clover extract, aqueous liquorice extract and nutmeg essential oil were prepared as described in a previous study [17].

The emulsion for lyophilization/spray-drying was prepared using maltodextrin, gum Arabic and inulin as excipients. The composition of the emulsion is given in Table 1.

Emulsion preparation: excipients were dissolved in liquorice extract and mixed with red clover extract then the essential oil was added. The liquid was homogenized with IKA^®^ ULTRA-TURRAX^®^ T18 homogenizer (IKA-Werke GmbH & Co., KG, Staufen, Germany) for 15 min, 3000 rpm. 

#### 2.2.1. Lyophilization Method Conditions

The emulsion (Table 1) required storage in the refrigerator for 48 h at 18 °C prior to lyophilization. For freeze-drying Beta 1-8 LSC plus lyophilizer (Martin Christ Gefriertrocknungsanlagen GmbH, Osterode am Harz, Germany) was equipped. Prior to the lyophilization process, the apparatus’ condenser was turned on for roughly 30 min to attain the necessary temperature (−53 °C). The product was lyophilized for 48 h. Throughout the article, microcapsules prepared using lyophilization will be named LM. 

#### 2.2.2. Spray-Drying Method Conditions

Spray drying was done using a Buchi B-291 Mini Spray-Dryer (BÜCHI Labortechnik AG, Flawil, Switzerland) under the following conditions: according to previous pilot studies inlet temperature was 160 °C, outlet temperature was 80–90 °C, spray flow feed rate—30 mL/min, air pressure—6 bar, aspirator—100%. Throughout the article, microcapsules prepared using spray-drying will be named SDM.

### 2.3. Evaluation of Physical Parameters of Microcapsules

The microcapsules obtained by spray-drying and lyophilization were analyzed for their product yield, moisture content, bulk and tapped volumes, solubility, and size distribution.

#### 2.3.1. Microcapsule Yields

The powder yield after spray-drying/lyophilization is given by the percentage ratio between the total mass of the final product by the non-solvent mass of the emulsion.

#### 2.3.2. Moisture Content

Using the Moisture Analyzer DAB (KERN & SOHN Gmb, Balingen, Germany), moisture content was determined. Microcapsules were weighed (approximately 0.2 ± 0.05 g) and heated at 105 °C until all the moisture was evaporated, and the product weight was constant. The device’s screen displayed the moisture content (%) after the procedure.

#### 2.3.3. Bulk and Tapped Volumes

The microcapsules’ compressibility index and Hausner ratio were evaluated using the TD 1 Tap Density Tester (SOTAX, Hopkinton, United States). The tapped density was obtained mechanically tapping a graduated measuring cylinder containing the powder sample. The Hausner ratio with compressibility index was calculated (values were shown on device’s screen). All measurements were carried out three times.

#### 2.3.4. Shape of the Microcapsules 

The morphological characteristics of the microcapsules produced with different technological methods were evaluated by optical microscopy, Eclipse 50i (Nikon, China). Small amounts of powders were placed on the surface of double-sided tape and 100× magnification was used.

#### 2.3.5. Solubility 

A total of 5.0 ± 0.05 g of the microcapsules were mixed with 30 mL of purified water for 30 min using a vortex mixer (IKA, Staufen, Germany). The solution was centrifuged at 1789× *g* for 20 min at 25 °C. After centrifugation, the sediment was transferred to pre-weighed Petri dishes and dried at 105 °C to constant weight. The solubility (%) of the microcapsules’ powder was calculated as the percentage of weight of sediment divided by the weight of the sample and multiplied by 100%.

#### 2.3.6. Size Distribution of Microcapsules

By using a laser, the volume weighted mean size (D 4,3) of the essential oil emulsions were calculated. Using the Mastersizer 3000 with the Hydro EV unit (Malvern Panalytical Ltd., Malvern, UK), diffraction size and distribution were evaluated. To achieve laser obscuration, samples were introduced drop-by-drop to the dispersant (water). The D10, D50, and D90 percentile values were used to describe the formulations.

### 2.4. Total Content of Active Compounds and In Vitro Release and Analysis of Microcapsules and Gel Tablets

#### 2.4.1. Total Phenolic and Flavonoid Content 

Total phenolic content was determined using the modified Slinkard et al. method [18]. A 0.5 mL volume of sample was mixed with 2.5 mL Folin–Ciocalteu’s phenol reagent (1:9 diluted in distilled water) and 2.0 mL of 7% (*w*/*v*) sodium carbonate. Absorbance was measured at 765 nm after 1 h using a spectrophotometer (Shimadzu UV-1800, Kyoto, Japan). The calibration curve was prepared using gallic acid. The results were expressed as gallic acid equivalents per gram dry weight (mg GA/g dw).

Flavonoid content was also determined using a slightly modified spectrophotometric method by Zhishen et al. [19]. Briefly, 0.1 mL of sample was added to 1.0 mL 96% (*v*/*v*) ethanol, 0.05 mL 33% acetic acid, 0.15 mL 10% aluminum chloride, and 2.0 mL 5% hexamethylenetetramine solutions. The samples were kept in the dark for 30 min and the absorbance was measured at a wavelength of 475 nm. The results were expressed as rutin equivalents per gram dry weight (RE/g dw).

#### 2.4.2. In Vitro Release of Active Compounds

In vitro release was performed in gastric and intestinal media as previously performed in the study of Matulyte et al. [20]. The research was achieved using the Sotax AT7 Smart Dissolution System (SOTAX AG, Aesch, Switzerland). Gastric medium was prepared according to the European pharmacopeia [21] using: 2.0 g of NaCl, 80 mL of 1 M HCl solution, 3.2 g of pepsin, and distilled water up to 1000 mL (pH = 1.2). 

Simulated intestinal juice was prepared using 6.8 g of KH_2_PO_4_, 77.0 mL of 0.2 M NaOH solution, 10 g of pancreas powder, and distilled water up to 1000 mL. The samples in gastric medium were incubated for 30–90 min and then moved to intestinal medium for 30–90 min. Samples for HPLC analysis were taken every half hour. The total in vitro release time interval was 0–180 min. The samples were filtered and prepared for analysis using headspace-gas chromatography with mass spectroscopy (HS-GC–MS) for volatile compounds from essential oil and high-performance liquid chromatography (HPLC) for isoflavones daidzein and genistein, and glycyrrhizin.

#### 2.4.3. Chromatographic Analysis of Microcapsules 

Nutmeg essential oil’s chemical compounds were determined using HS-GC–MS. Conditions and equipment used are described in a previous study by Matulyte et al. [22]. Quantitative analysis of volatile compounds was determined in microcapsules and chewable gel tablets.

The release of daidzein, genistein and glycyrrhizin were determined using high-performance liquid chromatography with diode array detectors (HPLC–PDA). The analysis was conducted using the Shimadzu Nexera X2 LC-30AD HPLC system (Shimadzu, Tokyo, Japan), equipped with an SPD-M20A diode array detector (DAD). The isoflavones’ research conditions are described in the previous work of Kazlauskaite et al. [17]. 

The mobile phase for glycyrrhizin detection consisted of solvent A (0.1% trifluoroacetic acid in water) and solvent B (acetonitrile). The linear gradient elution profile was as follows: 95% A/5% B at 0 min, 5% A/95% B at 30 min, 95% A/5% B at 36 min. The flow rate was 1 mL/min, and the injection volume was 10 μL. The chromatographic column was ACE C18 (250 × 4.6 mm) 5 µm, column storage temperature was 30 °C, UV/Vis range was 200 to 400 nm. The range of linearity of glycyrrhizin was 0.480 to 492 µg/mL. The contents were expressed as μg/g dry weight (dw). The calibration equation was y = 7210x + 9660, coefficient of determination (R^2^) was 0.9997. The limit of detection (LOD) was 0.095; limit of quantification (LOQ) was 0.42.

### 2.5. Antioxidant Activity of Microcapsules

The antioxidant activity of microcapsules and gel tablets were analyzed by spectrophotometric ABTS, DPPH and FRAP assays. ABTS, DPPH and FRAP methods were performed in the same as described in our previous study [17].

The ABTS method was initially reported by Miller and colleagues. It is based on the ability of an antioxidant to stabilize the ABTS colored cation radical, which can be previously formed by the oxidation of ABTS by methemoglobin and hydrogen peroxide [23]. Briefly, the test solution was prepared by mixing 2 mL of ABTS working solution with 200 μL of each test sample. The mixture was stored in a dark at room temperature for 30 min. The change in absorbance of the mixture was measured with a spectrophotometer at 734 nm. The calibration curve was obtained with a Trolox. The results were expressed as Trolox equivalents per gram dry weight (TE/g dw).

The DPPH method was first reported by M. Blois [24]. DPPH free radical scavenging is an accepted mechanism for screening antioxidant activity. The method used 2.0 mL of prepared DPPH solution mixed with 2.0 mL of the test samples. The reaction mixture was mixed and then incubated in the dark for 30 min. The absorbance was read at 517 nm. The calibration curve was obtained with a Trolox. The results were expressed as Trolox equivalents per gram dry weight (TE/g dw).

The FRAP assay was established by Benzie and Strain (1996) [25] and was used to determine the reducing activity in the plant raw material. The FRAP working reagent was prepared using 0.3 M acetate buffer, 10 mM TPTZ solution with 40 mM HCl, and 20 mM ferric chloride solution. A 10 μL amount of test sample was mixed with 200 μL of FRAP reagent and left in the dark. After 1 h, the absorbance of the mixture was measured at 593 nm. The calibration curve was obtained with ferrous sulphate. The results were expressed as ferrous sulphate equivalents per gram dry weight (FS/g dw).

### 2.6. Gel Tablets Preparation

#### 2.6.1. Gelatin Gel Tablets’ Preparation

Gelatin was poured with water and glycerol solution and left for 20 min to swell. In the porcelain dish apple juice, apple puree, and fruit powder were mixed. Gelatin was dissolved using a water bath (the temperature of gelatin mass was 50–60 °C) and the mass of the fruit was added to it. Microcapsules (5% *w*/*w*) were added in the gelatin gel mass, then poured into silicone molds and kept for 4 h in the refrigerator. After that gel tablets were taken out and kept in a plastic container at room temperature. The composition of gelatin gel tablets is given in Table 2. Although apple juice and apple puree were prepared in the laboratory, the products had no added sugar.

#### 2.6.2. Pectin Gel Tablets Preparation 

Five different compositions of pectin gel tablets were prepared (Table 3). First, apple juice, apple puree and other ingredients (except citric acid and pectin) were mixed and heated up to 40 °C. Pectin was added to the warm mass and dissolved. Next, mass was heated up to 100 °C and citric acid was added. Microcapsules (5% *w*/*w*) were added to the pectin gel mass, then poured into silicone molds and kept for 24 h at room temperature. After that pectin gel tablets were taken out and kept in a plastic container at room temperature.

### 2.7. Gel Tablets’ Physical Parameters Determination

Firmness, springiness, hardness, and stickiness were measured (n = 3) by a texture analyzer, Ta.XT.plus (Texture Technologies, New York, NY, USA). 

The parameters of texture were: return speed 10 mm/s; force 1 g; strain 50%; pre-test speed 1.00 mm/s; test speed 1.00 mm/s; post-test speed 10 mm/s; hold time 60 s; and trigger force 5.0 g.

### 2.8. Statistical Analysis

Data were analyzed using SSPS version 20.0 (IBM Corporation, Armonk, NY, USA). Physical parameters, antioxidant activity, total phenolic and flavonoid content experiments were performed three times. Data are expressed as mean ± standard deviation (S.D.). Comparisons between three different measurements were made using Friedman and Wilcoxon tests. In addition, comparisons between the two groups were made by the Mann–Whitney U test. The results were considered statistically significant at *p* < 0.05.

## 3. Results and Discussion

### 3.1. Preparation of Microcapsules and Physical Parameters Evaluation

Core material (red clover, liquorice extract and nutmeg essential oil) for spray-drying was obtained as explained in section “materials and methods”, using extraction techniques that have been developed previously to produce the largest numbers of isoflavones daidzein and genistein, saponin glycyrrhizin and main volatile monoterpenes found in nutmeg essential oil *α*-pinene, sabinene, *β*-myrcene and *β*-terpinene.

For lyophilization and spray drying the exact formulations of emulsions were used to prepare microcapsules. 

#### 3.1.1. Evaluation of Differently Prepared Microcapsules Yield, Moisture, and Quality 

After the production of microcapsules LM and SDM, the powder’s yield, moisture content and technological properties were evaluated (Table 4). The formulation of the microcapsules was the same.

The yield was 85.34% when preparing microcapsules using the lyophilization method and using spray-drying—45.12%. Results from the lyophilization method were much better. According to the findings, a severe buildup of the material on the cyclone walls of the spray dryer caused lower yields to be obtained by spray-drying than by lyophilization. Nevertheless, the moisture content in the SDM samples were significantly lower than LM’s. The moisture parameter is crucial because it can influence powder’s other physical parameters like flowability. According to published research, moisture content ought to fall below 4–5% and water activity should be below 0.20–0.25 to ensure stability [26]. Therefore, even though SDM moisture is lower than LM, it does not exceed the possible moisture limit.

The flowability of LM and SDM samples were different. According to compressibility index and Hausner ratio LM microcapsules’ powder was of fair quality, but SDM has very poor flowability (Table 4). Index’s and Hausner ratio values are presented in the Monograph of the European Pharmacopoeia [27]. Powders with higher moisture and irregular form have worse flowability [28]. In the pharmaceutical industry, powder flowability is a crucial parameter that affects manufacturing efficiency because of layer generation capabilities and content uniformity [29,30]. Consequently, preparing the powders flowability parameter should be a priority to produce quality product. 

**Figure 1:**
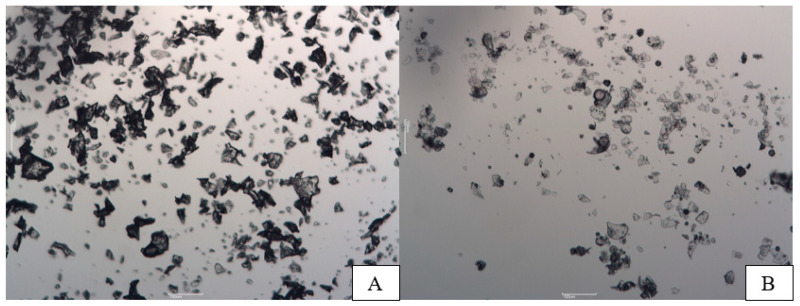
Structure of microcapsules (microscope magnification 100 times, (**A**)—LM microcapsules, (**B**)—SDM microcapsules).

The shape of LM and SDM microcapsule powders differed. Both samples’ microcapsules shapes and sizes were unequal. However, LM sample shapes were bigger compared with the SDM sample. Whereas spray-dried microcapsule powder featured small splinter-shaped particles and a spherical shape, freeze-dried microcapsule powder had an irregular form, like shattered glass (Figure 1). These irregularities in shape can be one of the reasons why powders did not have had excellent flowability.

#### 3.1.2. Microcapsules’ Solubility and Essential Oil Particles’ Distribution

Spray-dried microcapsules had a solubility of 95.16%, while lyophilized microcapsules had a solubility of 94.69%. Spray-dried microcapsules were more soluble, nevertheless, the differences were statistically insignificant (*p* > 0.05). It is possible that particle size affected solubility. 

Encapsulated essential oil (*Myristica fragrans* Houtt.) particle size and distribution were determined using a Mastersizer 3000. The particles produced using lyophilization and spray drying were 13.51 µm and 7.33 µm, respectively, expressed as D [4,3] (De Brouckere mean diameter).

**Figure 2:**
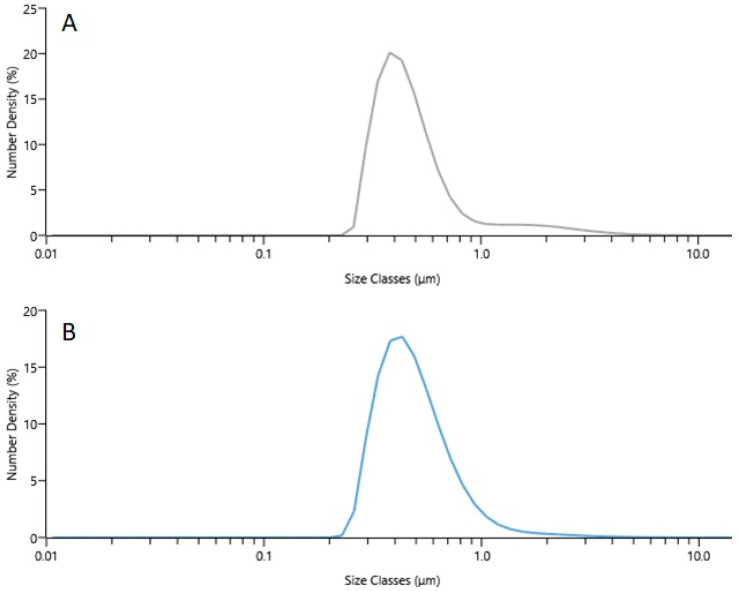
Essential oil particles’ distribution in microcapsules (**A**)—LM microcapsules, (**B**)—SDM microcapsules.

From the graph provided in Figure 2, part A, uniformity in essential oil is irregular in LM microcapsules, while in part B it is more even. Uniformity is a measure of absolute deviation from the median, LM sample uniformity was 0.533 and SDM 0.379. Therefore, LM essential oil particles’ sizes (in microcapsules) had a wider distribution range compared to the SDM samples. As mentioned before, this is influenced by the fact that the microcapsules obtained during lyophilization were broken glass-shaped, while spray-dried microcapsules were more rounded.

The distribution of essential oil drop percentiles in LM samples were—D10 was 0.319 ± 0.074 µm, D50 was 0.439 ± 0.053 µm, and D90 was 0.876 ± 0.066 µm. In SDM D10 was 0.317 ± 0.046 µm, D50 0.458 ± 0.065 µm and D90 0.796 ± 0.094 µm. These results revealed that in LM samples essential oil particles were bigger compared to SDM (Figure 2).

### 3.2. Antioxidant Activity of Active Compounds from Microcapsules and Release In Vitro

The manufacturing conditions of microcapsules can affect the active compounds. Severe cold or heat can harm them. Drying has been demonstrated to significantly alter the structure of various other antioxidant components and antioxidant activities, according to published research [31]. The high temperatures used in the microencapsulation technique can be damaging since essential oil molecules are heat sensitive, which may lead to deterioration or evaporation [32]. Nevertheless, the right use of the excipients in the production process can preserve antioxidant capacity and stabilize active compounds such as phenols, during microcapsules’ manufacturing [33,34].

#### 3.2.1. Antioxidant Activity of Microcapsules

Red clover extracts, that are used in both microcapsules’ formulation, contains phenols—isoflavones, which have been shown to have wide varieties of biological activities such as antibacterial, antiviral, antioxidant and anti-inflammatory [35]. Glycyrrhizin found in liquorice extract has been reported to possess anti-inflammatory, and antioxidant activities and can stimulate endogenous production of interferons [36]. These two extracts also contain a variety of other phenolic compounds, which may be lost to evaporation, thermal decomposition, or chemical and enzymatic degradation. According to studies, the main process causing the reduction in polyphenol concentration is due to the latter factors [37].

All the results obtained with LM samples were significantly higher compared to SDM samples obtained using spray-drying. Total phenolic content determined in the LM sample was 11.47 ± 0.01 mg GA/g dw and SDM 8.56 ± 0.42 mg GA/g dw. Therefore, the freeze-drying process was less harmful for phenols than spray-drying. These results agree with those reported in the literature; however, it was stated that both drying processes led to a partial loss of compounds like small molecular carbonyls, furans, and phenols [38]. In another study it was reported that freeze drying can generate a more significant amount of isoflavone powder from soybean cake than vacuum drying and spray drying [39].

Similar results for phenolic content was observed for total flavonoid content. LM results were higher compared to the same formulation sample SDM (Table 5). All the phenolic content results correlated with antioxidant activity determined using three different methods (DPPH, ABTS and FRAP). All the results obtained with the LM samples were statistically higher than SDM (*p* < 0.05). Comparing both samples’ preparation methods, antioxidant activity of samples prepared using freeze drying (LM) were 29.48% higher than SDM (ABTS method); the FRAP method resulted in LM being 31.34% higher than SDM; the DPPH method showed that LM samples were 40.45% higher. These results were similar to other research. In the study of Koffi et al., it was found that samples prepared using lyophilization and freeze-drying methods from Justicia secunda leaves’ polyphenols content and the antioxidant capacity of freeze-dried extract were higher than those of spray-dried extract. Additionally, freeze-dried powder gave better recovery yields [40].

#### 3.2.2. In Vitro Release of Active Compounds from Microcapsules

Since the lyophilized powder’s yield and encapsulation were more efficient than the spray-dried powder, further studies were performed with only LM microcapsules’ powder. The release was performed for 180 min (90 min in simulated gastric medium and 90 min in simulated intestine medium). Nevertheless, microcapsules fully dissolved after 30 min in intestine media, therefore, the results of main compound release in graphs are present until 120 min.

Although genistein has moderate intestinal absorption, larger doses could not be absorbed because of their poor solubility, necessitating the requirement of appropriate nutraceutical formulations. One of the main causes of genistein’s limited oral bioavailability is likely to be extensive metabolism [41]. However, orally administered genistein is more bioavailable than daidzein in foods and drinks. Regardless, different pharmacokinetics of daidzein have been reported. Still, these limitations of interpreting the findings relate to whether data from administration of pure compounds can really be translated to isoflavones contained within the food matrix [42,43,44].

It was suggested that glycyrrhizin from liquorice roots can be a potential bioactive constituent for managing oral diseases, when applied locally. However, it also can be administered orally, but it is metabolized to glycyrrhetinic acid by intestinal bacteria which contain β-D-glucuronidase, and that can cause side-effects as hypokalemia and hypertension [45,46].

The rate at which essential oils are required to be released from encapsulations differs depending on the application.

Microencapsulation protects volatile compounds from environmental exposure for a more extended period of time [47,48]. Spray-dried microcapsules with nutmeg essential oil did not lose their characteristic aroma when stored at room temperature for a month, and 4 active volatile compounds out of 11 (identified compounds in the essential oil) were identified [49]. The main volatile compounds identified in microcapsules (in this study) were: α-pinene, which modulates antibiotic resistance, by reducing the MIC value of ciprofloxacin, erythromycin, and triclosan, up to 512 times [50]; sabinene, which has anti-oxidant and anti-inflammatory properties and could be a potential compound for skeletal muscle atrophy prevention [51]; β-myrcene, which has potential analgesic, sedative, anti-diabetic, antioxidant, anti-inflammatory, antibacterial, and anticancer effects [52] and β-terpinene, which has an anti-inflammatory effect and inhibits the production of pro-inflammatory mediators [53].

**Figure 3:**
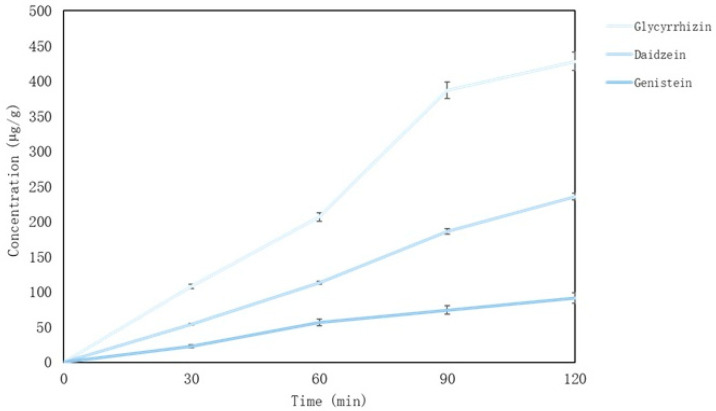
Main compounds (glycyrrhizin, daidzein and genistein) released in vitro from red clover and liquorice microcapsules.

After evaluating the release of the active compounds from microcapsules, it was found that microcapsules dissolve fully in intestinal medium after 60 min and glycyrrhizin, daidzein and genistein also dissolve. The concentrations of compounds after 120 min after release was 428.25 µg/mL, 235.63 µg/g and 91.36 µg/g (glycyrrhizin, daidzein and genistein, respectively) (Figure 3). The lowest concentration of all the compounds was genistein. In gastric medium, isoflavones, daidzein, and genistein were released gradually as well as in the intestinal medium. Regardless, glycyrrhizin release was most intense between 60 and 90 min (Figure 3).

**Figure 4:**
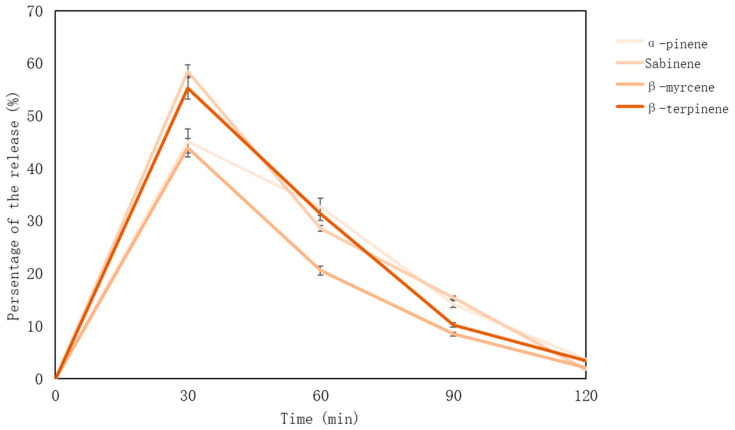
Monoterpenes’ release from nutmeg essential oil-loaded lyophilized microcapsules (%).

The percentage of monoterpenes is calculated based on the concentrations of compounds identified in the essential oil. The maximum number of essential oil compounds was determined after 30 min—later their amounts decrease. Sabinene is released the most—58.51% (after 30 min), the results are presented Figure 4.

The spray-drying method is more popular than lyophilization, the release of active compounds of nutmeg essential oil loaded microcapsules is determined for powders produced by the spray-drying method [49,54,55].

### 3.3. Gel Tablets Preparation and Physical Parameters’ Determination and Release in Simulated Media

Gelatin base chewable gel tablets were prepared based on previous studies’ results [22,56] and the microcapsules were added. For the pectin gel tablets, five compositions were modelled and one (C, Table 3) was chosen for the following research.

**Figure 5:**
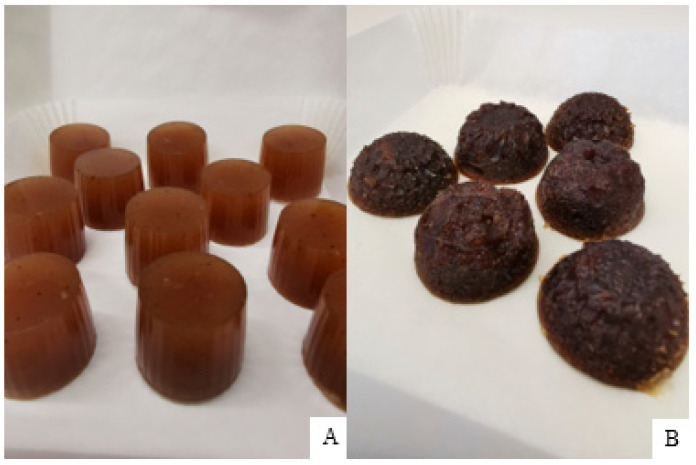
Gel tablets’ appearance (**A**) gelatin gel tablets; (**B**) pectin gel tablets.

The best composition was selected by the appearance and flavor of the tablets (E tablets were very hard, A and B were soft and were not taken out from the form without mass loss, and D lacked flavor intensity; compositions are shown in Table 3). Both tablets’ bases were prepared without added sugar. Gel tablets had appearance differences: gelatin tablets had smooth surface, while pectin was rough (Figure 5).

Citric acid was used as a flavor corrigent in gelatin base tablets, however, it was used as a gelification initiator [57] in pectin base tablets. HM pectin forms physical gels at pH < 3.5 [58]. Solid materials in the composition also influenced gel texture [59].

#### 3.3.1. Physical Parameters’ Comparisons of Gel Tablets with Different Bases

The texture of tablets is significantly affected by different polymers. Compared to pectin tablets, gelatin tablets were less firm and harder, but more elastic. Pectin base tablets with lyophilized microcapsules were firmest and hardest compared with other samples (Table 6). Microcapsules reduced the stickiness of gelatin base tablets by 68% after the addition of microcapsules.

Compared microcapsules’ influence on tablet texture was evaluated and microcapsules increased the firmness and hardness of tablets. Nevertheless, the stickiness decreased, and tablets were less sticky. The correlation of springiness was not determined, pectin tablets were less elastic than gelatin (compared with pectin and gelatin base control tablets, respectively).

Microcapsules’ tablets increased firmness and hardness (about 27% and 31%, respectively) in the pectin base, nevertheless, gelatin tablets were soft (about 63% and 34%, respectively).

In other studies, the firmness and springiness of chewable gel tablets with gelatin was 375.45 g and 74.09% (27% of the sugar was in composition) [56]. Gel tablets with 7% of gelatin and 70% of sweeteners springiness was 97.5% [60]. The texture results of gel tablets are compared with those of tablets of similar composition. It can be concluded that natural fruit powder significantly changed the texture of gel tablets.

Pectin base gummies had about 612 N of hardness (1.5% of pectin and 50% of sweeteners were in composition; 100 g of sample was pressed) [61]. It is too difficult to compare this study’s results with other data because a different texture test was applied and gel tablets with pectin are less popular than gelatin.

#### 3.3.2. Release of Active Compounds from Different Bases Gel Tablets

Two different methods evaluated the concentrations of active compounds. Isoflavones and glycyrrhizin were determined by HPLC and monoterpenes—HS-GC–MS.

**Figure 6:**
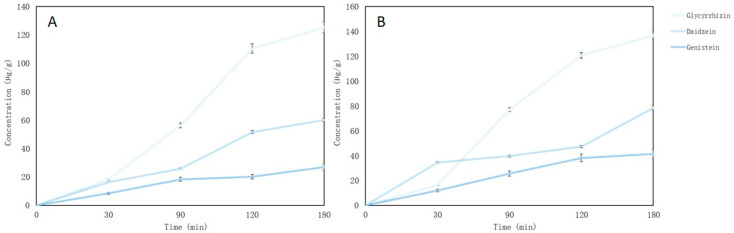
Isoflavones and saponin release from nutmeg essential oil-loaded lyophilized microcapsules (%); (**A**) gelatin gel tablets; (**B**) pectin gel tablets.

The highest concentrations of active compounds were determined in pectin base tablets: 9.1% of glycyrrhizin, 30.66% of daidzein and 53.32% of genistein. These compounds released gradually from the tablets and their amount increased depending on the duration of the study (Figure 6).

The last test sample was obtained after 360 min when the chewable tablets in the gut juice disintegrated. This test was performed to calculate the effectiveness of gel tablets. The effectiveness of the pectin tablet encapsulation was 71.36 ± 4.22% for glycyrrhizin, 72.03 ± 6.84% for daidzein and 64.83 ± 2.37% for genistein. In the gelatin tablet, active compounds’ release effectiveness was 63.41 ± 3.64% for glycyrrhizin, 61.78 ± 4.23% for daidzein and 60.11 ± 3.82% for genistein. The concentration of active compounds was lower in gelatin tablets compared to pectin. Incomplete relaxation of the compounds may be due to ballast materials (used puree, pectin, gelatin), temperature during production, and incomplete solubility of compounds in gut media.

**Figure 7:**
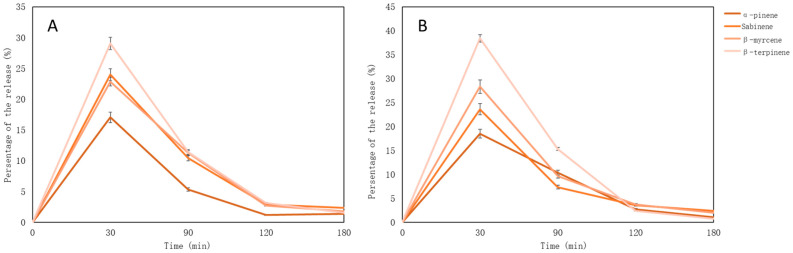
Monoterpenes’ release from nutmeg essential oil-loaded lyophilized microcapsules (%); (**A**) gelatin gel tablets; (**B**) pectin gel tablets.

Most of the monoterpenes were released after 30 min. (they are volatile compounds, so during a long experiment, their evaporation is not prevented) (Figure 7). The highest amount was of β-terpinene. The highest amount of compounds were released from pectin base tablets (*p* < 0.05) except for sabinene, as its maximum amount was released from the gelatin base (*p* > 0.05).

According to other studies, the maximum release of essential oil compounds was found after 30 min. (gelatin-based chewable gel tablets), however, other compounds had been identified [56]. The amount of volatile compounds from essential oils after 360 min was not investigated, because already after 60 min in gut media, the amount of compounds decreased strongly, and due to evaporation of the compounds, this study was not appropriate.

This technology with pectin based chewable tablets with lyophilized microcapsules can be applied in the development of pharmaceutical products. They can be adapted according to extracts and essential oils that are microencapsulated, and for biological activity (antiviral, antibacterial and antioxidant [17]). This technology is easily applicable to other essential oils and extracts, as the active compounds are preserved, including thermolabile compounds.

## 4. Conclusions

The microcapsules obtained by the lyophilization, and spray-drying methods showed different properties, although they were produced using the same formulation. Significant differences were found between the form, yield, and antioxidant activity. Lyophilized microcapsules had better properties, which led to their further use in the study.

The polymer Influenced tablet texture and active compounds' release. Chewable gelatin tablets were soft and elastic, while pectin tablets were hard, firm, and less sticky. Lyophilized microcapsules had a statistically significant influence on tablet parameters compared with control pectin tablets. Active compounds were released in larger quantities from pectin-based tablets. There was no statistically significant difference in the amount of compounds released in the gastric and intestinal media, the release of the compounds was significantly affected by the duration of the study. The method and formulations of freeze-dried microcapsules in pectin or gelatin base are appropriate for use in the pharmaceutical or food industry for effective flavor (with no added sugar) or active substances’ release during oral administration. Additionally, the chewable tablet with lyophilized microcapsules technology is easily applicable for other substances sensitive to environmental factors.

## Figures and Tables

**Table 1:** Emulsion composition.

Ingredient	Amount (%)
Red clover extract	12.4
Liquorice extract	49.59
Nutmeg essential oil	0.83
Gum Arabic	5.58
Maltodextrin	18.6
Inulin	13.2

**Table 2:** Gelatin gel tablets composition.

Ingredient	Amount (%)
Apple juice	38.8
Apple puree	14.9
Banana powder	20.7
Gelatin	7.3
Water	10.3
Glycerol	8.0

**Table 3:** Pectin gel tablets’ composition.

Ingredient	Sample				
	A	B	C	D	E
	Amount (%)				
Apple juice *	76.2	65.2	46.1	40.4	-
Apple puree *	20.3	25.3	41.4	23.2	55.7
Blackcurrant puree *	-	-	9.5	-	-
Buckthorn puree *	-	6.3	-	-	-
Banana powder	-	-	-	-	29.3
Lingonberry powder	-	-	-	15.4	-
Pectin	2.0	1.7	1.5	2.0	2.0
Citric acid 50% solution	1.5	1.5	1.5	1.5	2.0
Water	-	-	-	17.5	11.5

* Ingredients were prepared in the laboratory, the products had no added sugar.

**Table 4:** Physical microcapsules’ parameters.

Samples	Yield, %	Moisture, %	Carr Index, %	Hausner Ratio Value
LM	85.34 ± 3.17 *	4.45 ± 0.23 *	20.83	1.263
SDM	45.12 ± 7.81	3.24 ± 0.36	38.71 *	1.632 *

* The difference between samples’ parameters statistically significant at *p* < 0.05.

**Table 5:** Total phenolics, flavonoids content and antioxidant activity of microcapsules.

Sample	Total Phenolic Content, mg GA/g dw	Total Flavonoids Content, mg RU/g dw	DPPH, µg TE/g dw	ABTS, µg TE/g dw	FRAP, mg FS/g dw
LM	11.47 ± 0.01 *	7.56 ± 0.02 *	8.85 ± 0.01 *	136.73 ± 0.73 *	252.62 ± 0.01 *
SDM	8.56 ± 0.42	5.21 ± 0.22	5.27 ± 0.02	96.41 ± 0.93	173.25 ± 0.25

* The difference between samples parameters statistically significant at *p* < 0.05.

**Table 6:** Chewable gel tablets texture properties depending on the base.

Samples	Mass of the Sample	Firmness (g)	Stickiness (g)	Hardness (g)	Springiness (%)
P ^a^	5.51 ± 0.67	1027.8 ± 179.04 ***	−24.39 ± 1.85 ***	609.3 ± 233.03 ***	41.79 ± 0.74 ***
PLM	6.04 ± 0.58	1303.3 ± 377.28 *	−27.25 ± 15.15	798.09 ± 201.12	38.38 ± 0.23 *
G ^b^	5.73 ± 0.25	895.12 ± 62.78	−20.23 ± 5.92	738.27 ± 27.24	64.58 ± 1.35
GLM	5.77 ± 0.36	548.69 ± 12.63 **	−34.04 ± 2.91 **	549.51 ± 11.36 **	68.02 ± 2.53

^a^—control pectin base gel tablet, ^b^—control gelatin base tablet. * *p* < 0.05 versus pectin base tablets, ** *p* < 0.05 versus gelatin base tablets, *** *p* < 0.05 versus gelatin base tablet.

## Data Availability

Data is contained within the article.
